# Expression profiling of circulating lncRNA GIAT4RA, lncRNA AATBC, lncRNA Sirt1-AS, and SMARCB1 in lung cancer patients

**DOI:** 10.1186/s12885-024-12896-1

**Published:** 2024-09-23

**Authors:** Neveen A. Hussein, Samia A. Ebied, Abdel Aziz M. Belal, Mohamad A. Ahmad, El Sayed A. Weheida

**Affiliations:** 1 Applied Medical Chemistry Department, Medical Research Institute, Alexandria University, Alexandria, Egypt; 2 Clinical Oncology and Nuclear Medicine, Faculty of Medicine, Alexandria University, Alexandria, Egypt; 3 Clinical Pathology Department, Military Medical Academy, Cairo, Egypt

**Keywords:** Lung cancer, lncRNA GIAT4RA, lncRNA AATBC, lncRNA Sirt1-AS, SMARCB1

## Abstract

Long noncoding RNAs (lncRNAs) are crucial regulators of biological processes such as transcription interference and activation, chromatin remodeling, and mRNA translation. Uncontrolled gene expression could result from various epigenetic modifiers, like lncRNAs. So, this study aimed to evaluate the expression profiles of lncRNA GIAT4RA, lncRNA AATBC, lncRNA Sirt1-AS, and SMARCB1 in lung cancer. The current study included lung cancer patients (*n* = 50), patients with chronic inflammatory diseases (*n* = 30), and healthy volunteers (*n* = 20). The expression of blood genes and the concentration of serum neuron-specific enolase were determined by real-time PCR and electrochemiluminescence immunoassay, respectively. The receiver operating characteristic and Kaplan-Meier analyses assess the sensitivity of genes as diagnostic and prognostic biomarkers, respectively. LncRNA GIAT4RA and lncRNA AATBC were upregulated, while lncRNA Sirt1-AS was significantly downregulated in all patients compared to the control group. SMARCB1 expression was significantly downregulated in chronic inflammatory patients, while in those with lung cancer, it showed an insignificant difference. The expression of lncRNA GIAT4RA and lncRNA AATBC was significantly related to the stage of lung cancer. The survival analyses showed that lower lncRNA Sirt1-AS was linked to lung cancer patients’ poorer disease-free survival and overall survival. Differences in lncRNA GIAT4RA, lncRNA AATBC, and lncRNA Sirt1-AS expression were detected in all patients. The consequent abnormal expression of lncRNAs could be crucial in lung cancer development. LncRNA GIAT4RA, lncRNA AATBC, and lncRNA Sirt1-AS may be utilized as promising diagnostic biomarkers. LncRNA AATBC, lncRNA Sirt1-AS, and SMARCB1 may be valuable prognostic biomarkers for lung cancer.

**Supplementary Information:**

The online version contains supplementary material available at 10.1186/s12885-024-12896-1.

## Introduction

Approximately 2.2 million new cases and 1.8 million deaths from lung cancer occur each year worldwide, making it the most lethal malignancy for both men and women [1]. Non-small cell lung cancer (NSCLC, 85%) and small cell lung cancer (SCLC, 15%) are two different types of lung cancer. The cancer stage at the time of diagnosis is one of the key indicators of survival, and whereas localized disease in NSCLC has a 5-year survival rate of 57.4%, distant metastatic disease has an exceedingly low 5-year survival rate of just 5.2% [2].

LncRNAs are essential regulators of gene expression and genome structure, and they have recently received a lot of interest due to their roles during growth and illnesses like cancer. Although research on cancer epigenetic factors and oncogenes has found several epigenetic modifiers, like lncRNAs, which have a role in the development of different cancers [3], lncRNAs can interact directly with nucleosome-remodeling molecules and chromatin-modifying enzymes to regulate chromatin structure and the accessibility of genetic material for gene suppression [4].

LncRNA GIAT4RA (LOC102723729) is found in the region between GINS4 (chromosome 8p11.21) and GPAT4. The chromatin modifier lymphoid-specific helicase is degraded by lncRNA GIAT4RA [5]. LncRNA AATBC (apoptosis-associated transcript in bladder cancer, LOC284837) was first identified in bladder cancer, and its down-regulation induced apoptosis and repressed proliferation in bladder cancer [6]. Tang et al., also discovered that lncRNA AATBC facilitated nasopharyngeal cancer development and metastasis [7].

LncRNA Sirt1 antisense (lncRNA Sirt1-AS) is a natural antisense transcript RNA (758 bp) that has the same gene locus as Sirt1 3’untranslated region (3’-UTR) and has a fully overlapping complementary sequence from tail to tail by the principle of base complementary pairing [8]. LncRNA Sirt1-AS could attach to and completely overlap with the 3’-UTR of Sirt1 mRNA, increasing Sirt1 mRNA stability by producing a lncRNA-mRNA duplex, and impacting Sirt1 expression [9]. Accumulated evidence suggests that lncRNA Sirt1-AS contributes to a variety of disorders by enhancing the abundance of Sirt1 [8].

Switch/sucrose non-fermentable (SWI / SNF)-related matrix-associated actin-dependent regulator of chromatin, subfamily B, member 1 [SMARCB1, known as integrase interactor 1 (INI1)] is one of the core subunits of the SWI / SNF complex. SMARCB1 controls gene expression and regulates transcriptional processes by participating in chromatin remodeling and the continuous change of chromatin structure [10].

As a result, this study assesses the expression profiles of lncRNA GIAT4RA, lncRNA AATBC, lncRNA Sirt1-AS, and SMARCB1 genes in lung cancer patients. Moreover, the correlations of these genes with the clinicopathological traits of lung cancer patients were carried out.

## Subjects and methods

### Patients

The current study included healthy volunteers (*n* = 20; range: 38–81; mean: 59.05 years), non-cancer patients with chronic inflammatory diseases (bronchial asthma and obstructive pulmonary diseases) (*n* = 30; range: 40–78; mean: 59 years) who were chosen from Sadr El Mamoura Hospital, and newly diagnosed lung cancer patients (*n* = 50; range: 34–86; mean: 57.2 years) who were chosen from those admitted to Clinical Oncology and Nuclear Medicine Department, Faculty of Medicine, Alexandria.

All lung cancer patients met the following requirements: primary invasive lung carcinoma, no signs of infection, no immunosuppressive medication or chemotherapy, and no blood transfusions during the previous three weeks. The following investigations were done for lung cancer patients: full history recording, preoperatively fine needle aspiration cytology of the lung mass, routine laboratory investigations (complete blood picture, bleeding, and coagulation times), and radiological investigations including x-ray chest, and CT scan [11]. This study has been approved by the Medical Research Institute Ethics Committee (E / C. S / N. T37/2019), Alexandria University, and according to the Declaration of Helsinki. Informed consent was obtained from all participants.

### Sampling

Before beginning any treatment, three ml of blood samples were collected from patients and controls in tubes containing K_3_EDTA and stored at -80 ^o^C until used for the quantification of lncRNA GIAT4RA, lncRNA AATBC, lncRNA Sirt1-AS, and SMARCB1 by real-time PCR. Moreover, two ml of blood were collected in a tube without anticoagulant and centrifuged at 6000 rpm for 10 min. Serum samples were stored at -20 ^o^C for neuron-specific enolase (NSE) determination by electrochemiluminescence immunoassay.

### Quantification of lncRNA GIAT4RA, lncRNA AATBC, lncRNA Sirt1-AS, and SMARCB1 expression

The miRNeasy Mini Kit (Qiagen, Germany) was used to extract total RNA from 200 µl whole blood samples. QIAzol lysis reagent, RWI buffer, RPE buffer, and RNAase-free water were used for RNA extraction. The concentrations of RNA were confirmed in each sample by measuring at 260 nm (NanoDrop spectrophotometer).

Template RNA, 4 µl RT buffer, 2 µl dNTP mix, 1 µl reverse transcriptase enzyme, 1 µl RNase inhibitor, 1 µl random hexamer primer, and nuclease-free water were used for reverse transcription of RNA by RevertAidTM First Strand cDNA Synthesis Kit (Thermo Fisher Scientific). The primers (Invitrogen, Life Technologies) and Maxima SYBR Green qPCR Master Mix were used to measure the expression of lncRNA GIAT4RA, lncRNA AATBC, lncRNA Sirt1-AS, SMARCB1 and glyceraldehyde 3-phosphate dehydrogenase (GAPDH, housekeeping gene) Table 1. The thermal cycling was 95 ◦C for 10 min followed by 40 cycles 95 ◦C for 15 s, annealing is variable (55 ◦C for lncRNA GIAT4RA, lncRNA AATBC, SMARCB1 and 45 ◦C for lncRNA Sirt1-AS) for 30 s, the extension at 72 ◦C for 30 s. The final extension was 1 cycle at 72 ◦C for 5 min. The 2^−ΔΔCt^ was applied to express all investigated genes [12] (Supplementary Fig. 1A-E).

**Table 1:** Primer sequences

Genes	Primers
**LncRNA GIAT4RA**	Forword 5ʹTTGGTGGATGCTGACCTTCA3ʹ
	Reverse 5ʹACCAGAAACCAGATGTAGCCA3ʹ
**LncRNA AATBC**	Forword 5ʹCTGCTTCTCCAGGTCTCTTTCC3ʹ
	Reverse 5ʹCACAGGGACATTGAGTGAAGGC3ʹ
**LncRNA Sirt1-AS**	Forword 5ʹTCTGCTATTACAAGTTACAT3′
	Reverse 5ʹCCTGATTATACAGTTCCAA3′
**SMARCB1**	Forword 5′GGCATCAGAAGACCTACGCCTT3′
	Reverse 5′CTCCATCTCAGCGTCTGTCAGA3′
**GAPDH**	Forword 5′GTCTCCTCTGACTTCAACAGCG3′
	Reverse 5′ACCACCCTGTTGCTGTAGCCAA3′

### NSE concentration (ng/ml)

Electrochemiluminescence immunoassay (ECLIA) on the Cobas-e immunoassay analyzer was used to determine the NSE protein [13].

### Statistical analyses

All data has been statistically analyzed using IBM SPSS software package, version 20.0. Kolmogorov-Smirnov was used to test the normality of distributions of quantitative variables. Kruskal-Wallis test, a pairwise comparison between every two groups was done using the Post Hoc test (Dunn’s for multiple comparisons). The spearman test was used for correlations. Receiver operating characteristic curve (ROC) was plotted to evaluate a suggested cut-off. The test’s diagnostic performance is indicated by the area under the ROC curves. The Kaplan-Meier method was utilized to compute disease-free survival (DFS) and overall survival (OS) curves. Statistical significance was considered at P < 0.05.

## Results

Table 2 illustrates the characteristics of the control group, patients with chronic inflammatory diseases, and patients with lung cancer. According to histopathological type, 39 lung cancer patients were NSCLC, and 11 were SCLC. Regarding the stages of NSCLC, only one patient was stage II, 6 patients were stage III, and 32 patients were stage IV. For SCLC stages, only one patient was stage II, 2 patients were stage III, and 8 patients had stage IV.

**Table 2:** Characteristics of control, and patients

	Control *n* (%)	Chronic inflammatory *n* (%)	Lung cancer *n* (%)
**Gender**			
Male	12 (60)	21 (70)	44 (88)
Female	8 (40)	9 (30)	6 (12)
**Age (years)**			
< 40	1 (5)		6 (12)
≥ 40	19 (95)	30 (100)	44 (88)
**Smoking**			
Yes	12 (60)	20 (66.7)	44 (88)
No	8 (40)	10 (33.3)	6 (12)
**Family history**			
Negative			36 (72)
Positive			14 (28)
**Tumor size**			
< 5			16 (32)
> 5			34 (68)
**Lymph node metastasis**			
Negative			8 (16)
Positive			42 (84)
**Histopathology of lung cancer**			
NSCLC			39 (78)
SCLC			11 (22)
**Stages of NSCLC**			
II			1 (2)
III			6 (12)
IV			32 (64)
**Stages of SCLC**			
II			1 (2)
III			2 (4)
IV			8 (16)
**Pathological grade**			
I (well differentiation)			7 (14)
II (moderate differentiation)			20 (40)
III (poorly differentiation)			23 (46)
**NSE (ng/ml)**			
< 16.3	20 (100)	30 (100)	14 (28)
> 16.3			36 (72)
**CEA**			
< 5	20 (100)	26 (86.7)	15 (30)
> 5		4 (13.3)	35 (70)

*NSCLC* Non-small cell lung cancer, *SCLC* Small cell lung cancer, *NSE* Neuron-specific enolase, *CEA* Carcinoembryonic antigen

### LncRNA GIAT4RA, lncRNA AATBC, lncRNA Sirt1-AS, SMARCB1 expression, NSE concentration and their correlations

**Fig. 1:**
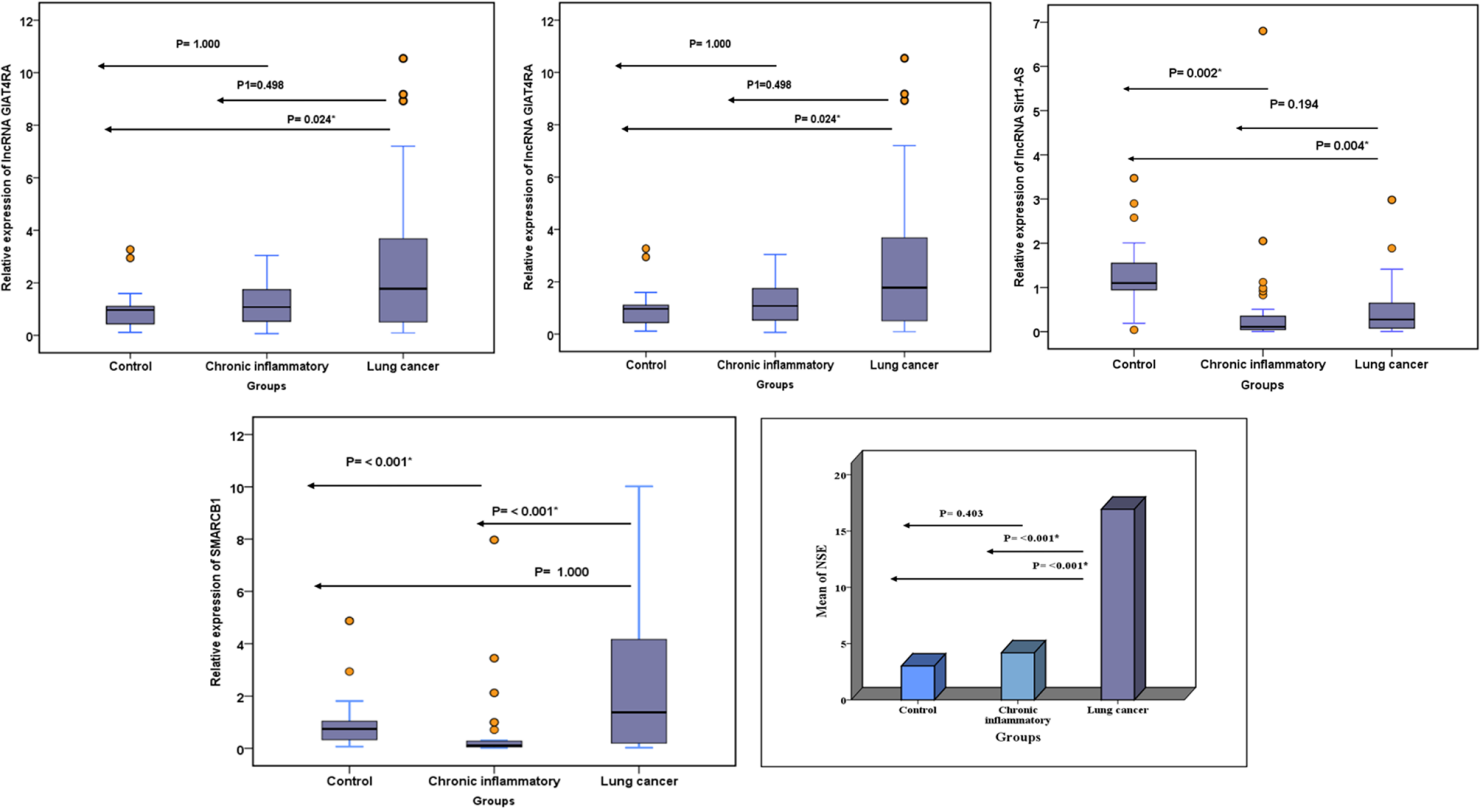
LncRNA GIAT4RA, lncRNA AATBC, lncRNA Sirt1-AS, SMARCB1 expression, and NSE concentration in control and patients. * P < 0.05

For chronic inflammatory patients, lncRNA AATBC expression was significantly upregulated (*P* = 0.002), while the expression of lncRNA Sirt1-AS and SMARCB1 was significantly downregulated (*P* = 0.002, < 0.001, respectively), and lncRNA GIAT4RA showed insignificant differences (*P* = 1.000) compared to the control group. In lung cancer patients, the relative expression of lncRNA GIAT4RA and lncRNA AATBC was significantly upregulated (*P* = 0.024, 0.001, respectively), while lncRNA Sirt1-AS was significantly downregulated (*P* = 0.004), and SMARCB1 showed insignificant differences (*P* = 1.000) compared to the control group. Additionally, studied lncRNAs had insignificant differences (*P* = 0.498, 1.000, 0.194, respectively) whereas SMARCB1 was significantly upregulated (P < 0.001) compared to chronic inflammatory patients (Fig. 1).

NSE values in lung cancer were statistically higher than in the chronic inflammatory or control groups (*P* < 0.001). When comparing the NSE concentration of chronic inflammatory patients with the control group, there was no significant difference (*P* = 0.403) (Fig. 1).

**Fig. 2:**
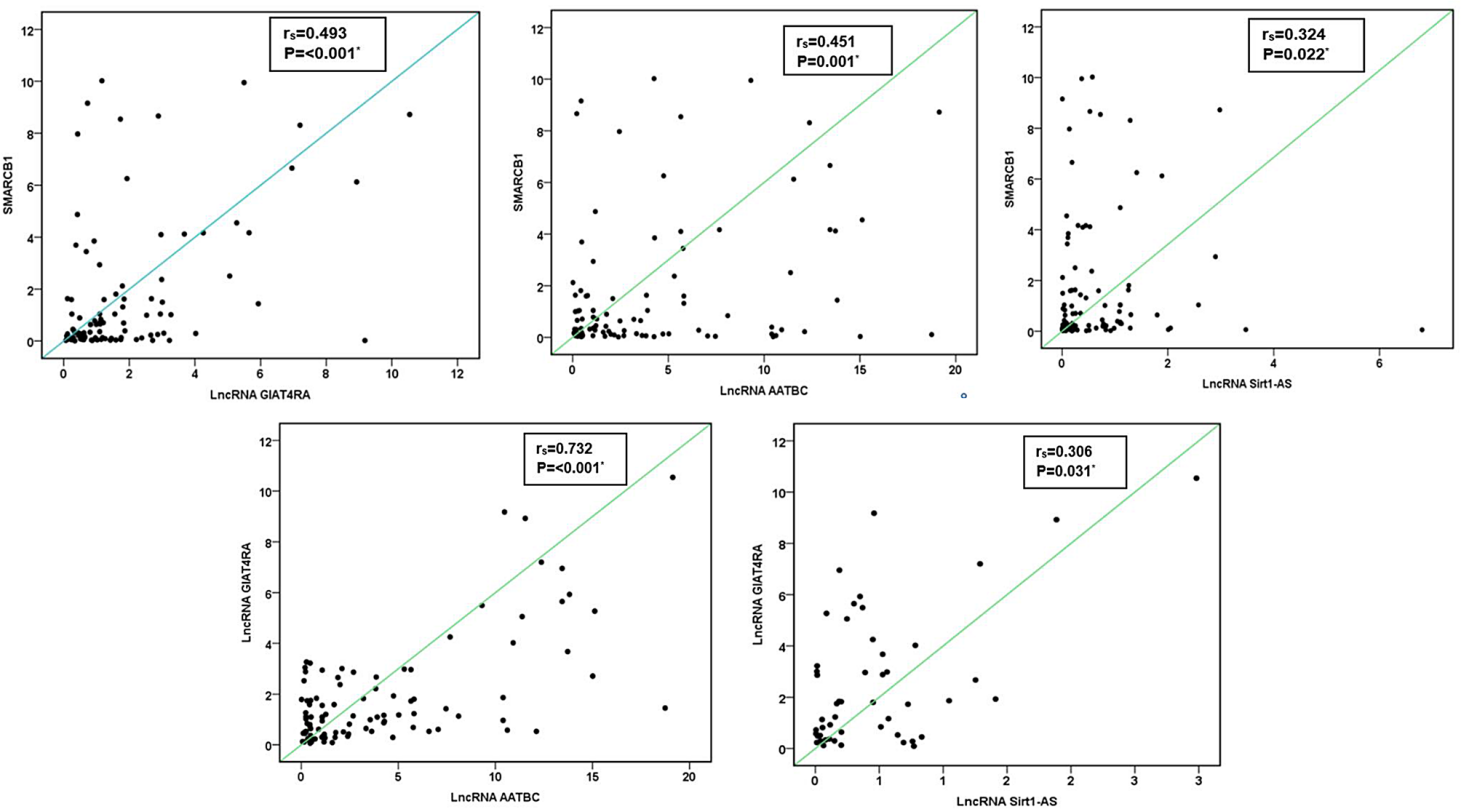
LncRNA GIAT4RA, lncRNA AATBC, lncRNA Sirt1-AS, and SMARCB1 correlations in lung cancer patients

In patients with chronic inflammatory disease, none of the studied parameters showed any correlation with each other. On the other hand, patients with lung cancer had statistically positive correlations between each of lncRNA GIAT4RA, lncRNA AATBC, lncRNA Sirt1-AS, and SMARCB1 except between lncRNA AATBC, and lncRNA Sirt1-AS (*P* = 0.074). No correlations were observed between all gene expressions and NSE concentration (Fig. 2). CEA was correlated with lncRNA AATBC expression only (*P* = 0.006) in patients with chronic inflammatory disease (Supplementary Table 1).

### LncRNA GIAT4RA, lncRNA AATBC, lncRNA Sirt1-AS, SMARCB1, NSE relations with patients’ characteristics

**Fig. 3:**
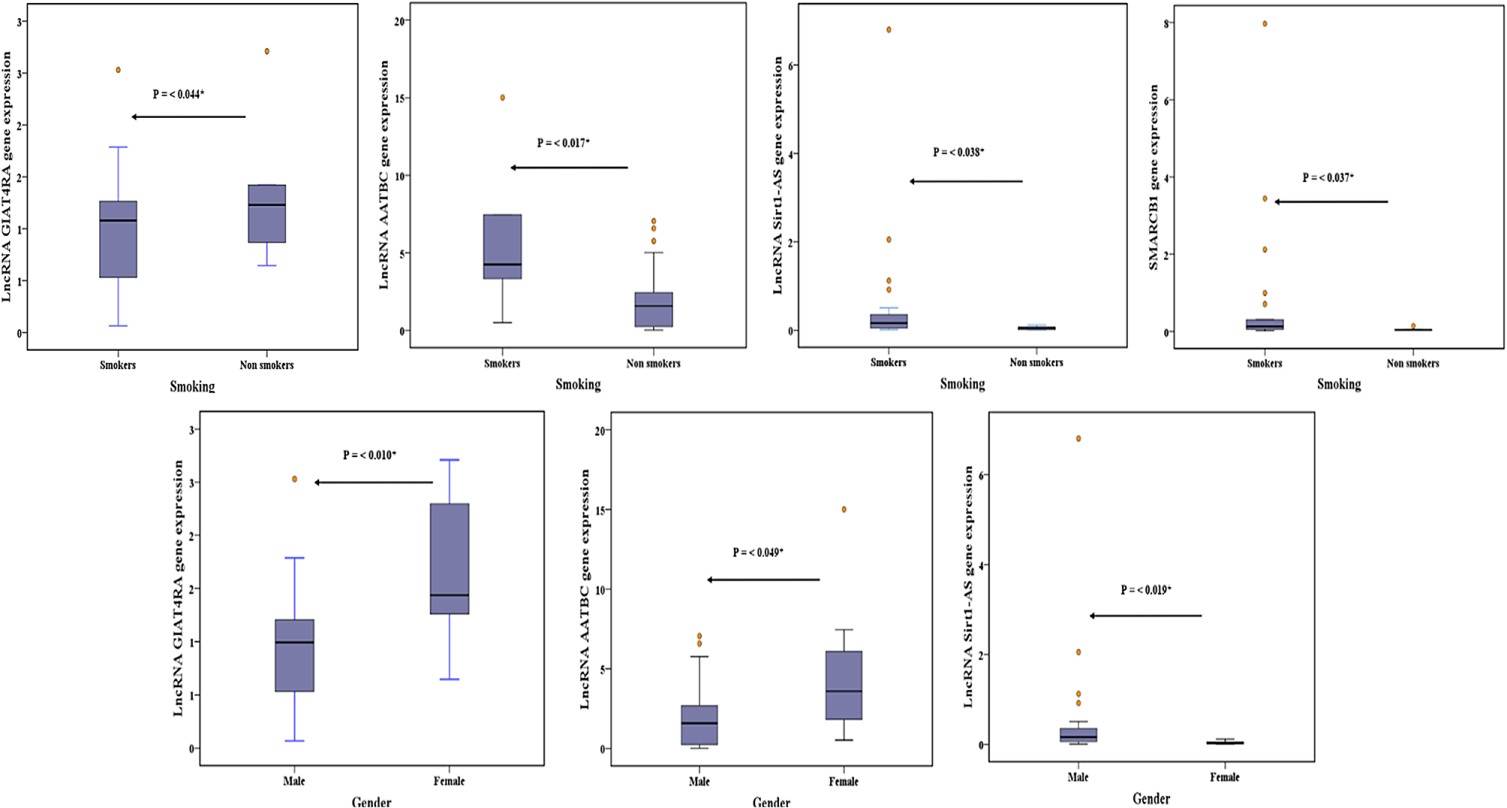
LncRNA GIAT4RA, lncRNA AATBC, lncRNA Sirt1-AS, and SMARCB1 relations with smoking and gender of chronic inflammatory patients

In patients with chronic inflammatory disease lncRNA GIAT4RA, lncRNA AATBC, lncRNA Sirt1-AS were related to gender (*P* = 0.01, 0.049, 0.019, respectively) and smoking (*P* = 0.044, 0.017, 0.038, respectively). SMARCB1 was related to smoking only (*P* = 0.037) (Fig. 3, Supplementary Table 2).

**Fig. 4:**
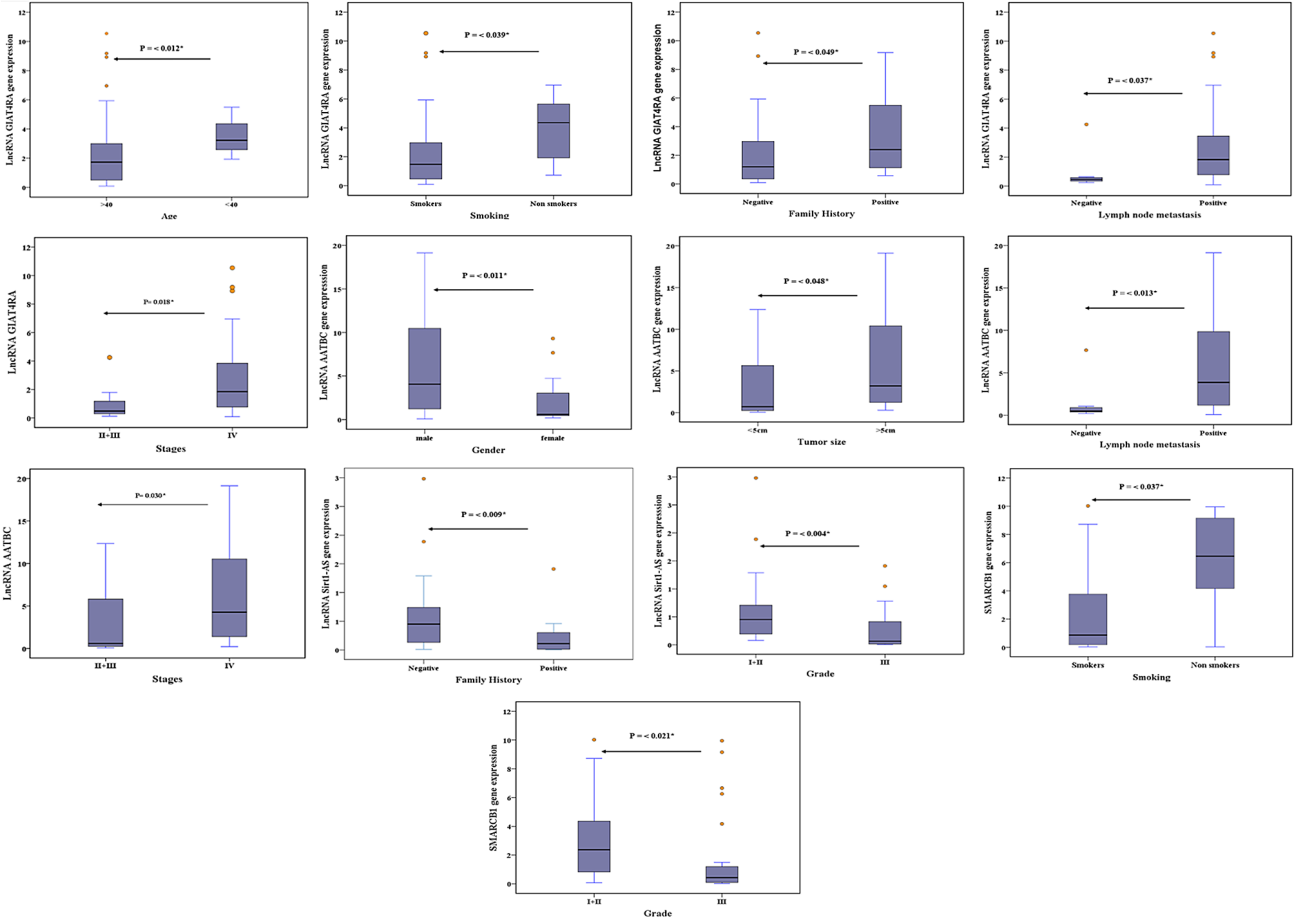
LncRNA GIAT4RA, lncRNA AATBC, lncRNA Sirt1-AS, and SMARCB1 relations with characteristics of lung cancer patients

In lung cancer patients lncRNA GIAT4RA was related to age (*P* = 0.012), smoking (*P* = 0.039), family history (*P* = 0.049), lymph node metastasis (*P* = 0.037), and stage (*P* = 0.018). LncRNA AATBC was related to gender (*P* = 0.011), tumor size (*P* = 0.048), lymph node metastasis (*P* = 0.013), and stage (*P* = 0.030). LncRNA Sirt1-AS was related to grade and family history (*P* = 0.004, 0.009, respectively), while SMARCB1 was related to smoking and grade (*P* = 0.037, and 0.021) (Fig. 4, Supplementary Table 2).

### ROC, DFS, and OS analyses

**Fig. 5:**
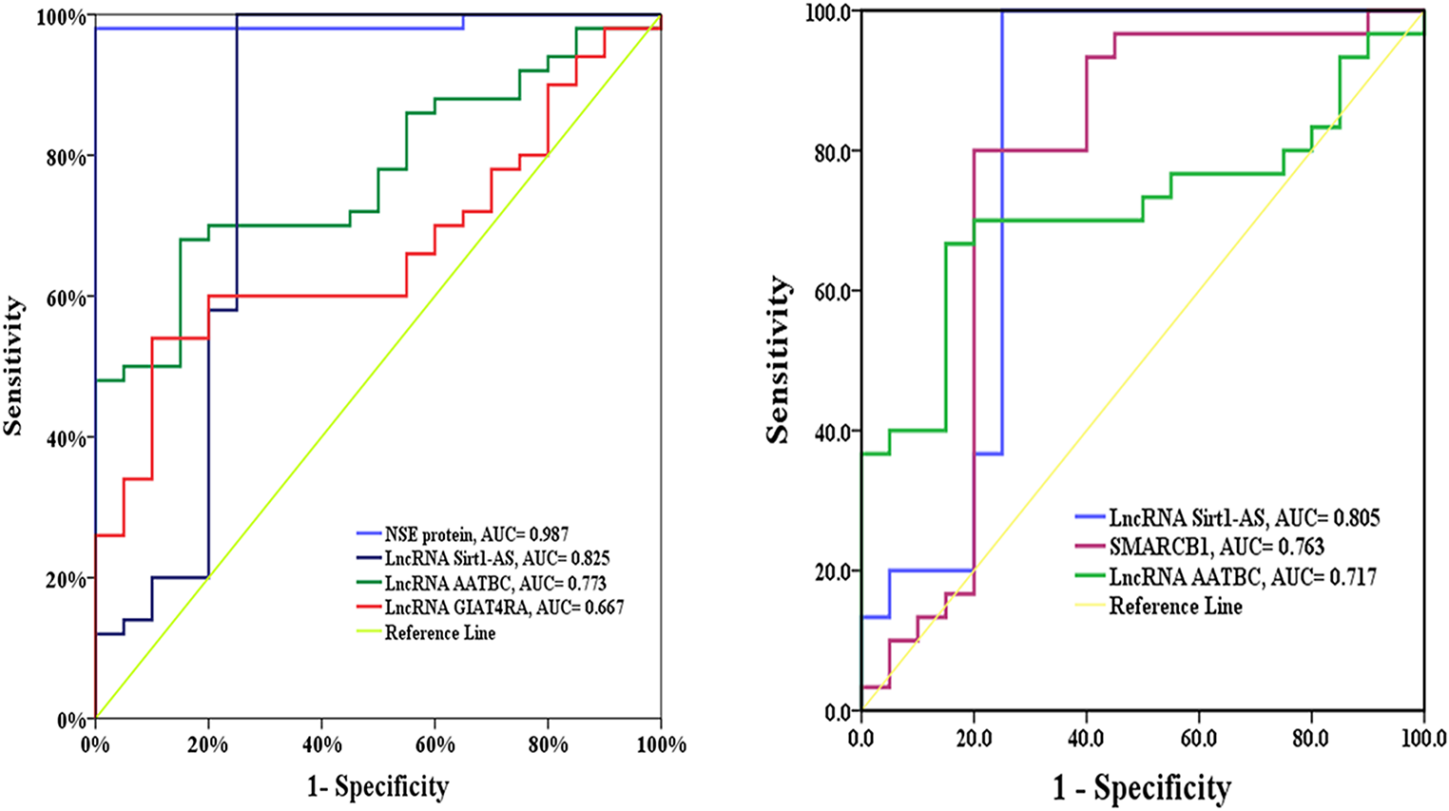
ROC curves for lncRNA GIAT4RA, lncRNA AATBC, lncRNA Sirt1-AS, SMARCB1 and NSE protein in (**A**) lung cancer, and (**B**) chronic inflammatory patients

In lung cancer patients, lncRNA GIAT4RA, lncRNA AATBC, lncRNA Sirt1-AS, and NSE had a higher area under the ROC curve (66.7, 77.3, 82.5, 98.7%, respectively, *P* = 0.030, <0.001) with sensitivity (60, 70, 90, 98%, respectively) and specificity (80, 80, 75, 100%, respectively). In patients with chronic inflammatory disease, lncRNA AATBC, lncRNA Sirt1-AS, and SMARCB1 had a higher area under the ROC curve (71.7, 80.5, 76.3%, respectively, *P* = 0.010, <0.001, 0.002) with sensitivity (70, 90, 80%, respectively) and specificity (80, 75, 80%) (Table 3; Fig. 5).

**Table 3:** The area under ROC curves, sensitivity, and specificity for lncRNA GIAT4RA, lncRNA AATBC, lncRNA Sirt1-AS, SMARCB1, and NSE

		Control (*n* = 20)	Patients	Sensitivity	Specificity	AUC	NPV	PPV	Accuracy	*P*
		**Lung cancer patients (** ***n*** ** = 50)**								
**LncRNA GIAT4RA**	≤ 1.11	15	20	60	80	66.7	80	60	65.7	0.030*
	> 1.11	5	30							
**LncRNA AATBC**	≤ 1.09	16	15	70	80	77.3	80	70	72.8	< 0.001*
	> 1.09	4	35							
**LncRNA Sirt1-AS**	≤ 1.06	5	45	90	75	82.5	75	90	85.7	< 0.001*
	> 1.06	15	5							
**NSE**	≤ 8.38	20	3	98	100	98.7	100	93.9	95.71	< 0.001*
	> 8.38		47							
		**Chronic inflammatory patients (** ***n*** ** = 30)**								
**LncRNA AATBC**	≤ 1.12	16	9	70	80	71.7	84	64	74	0.010*
	> 1.12	4	21							
**LncRNA Sirt1-AS**	≤ 0.01	15	3	90	75	80.5	83.3	84.3	84	< 0.001*
	> 0.01	5	27							
**SMARCB1**	≤ 0.042	15	5	80	80	76.3	85.7	72.7	80	0.002*
	> 0.042	5	25							

*NPV* Negative predictive value, *AUC* Area under the curve, *PPV* Positive predictive value, *NSE* Neuron-specific enolase

**Fig. 6:**
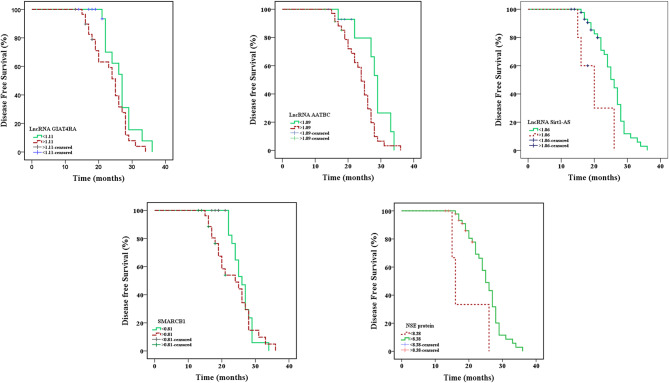
Kaplan-meier survival curves for disease free survival of lncRNA GIAT4RA, lncRNA AATBC, lncRNA Sirt1-AS, SMARCB1 expression, and NSE in lung cancer patients

**Fig. 7:**
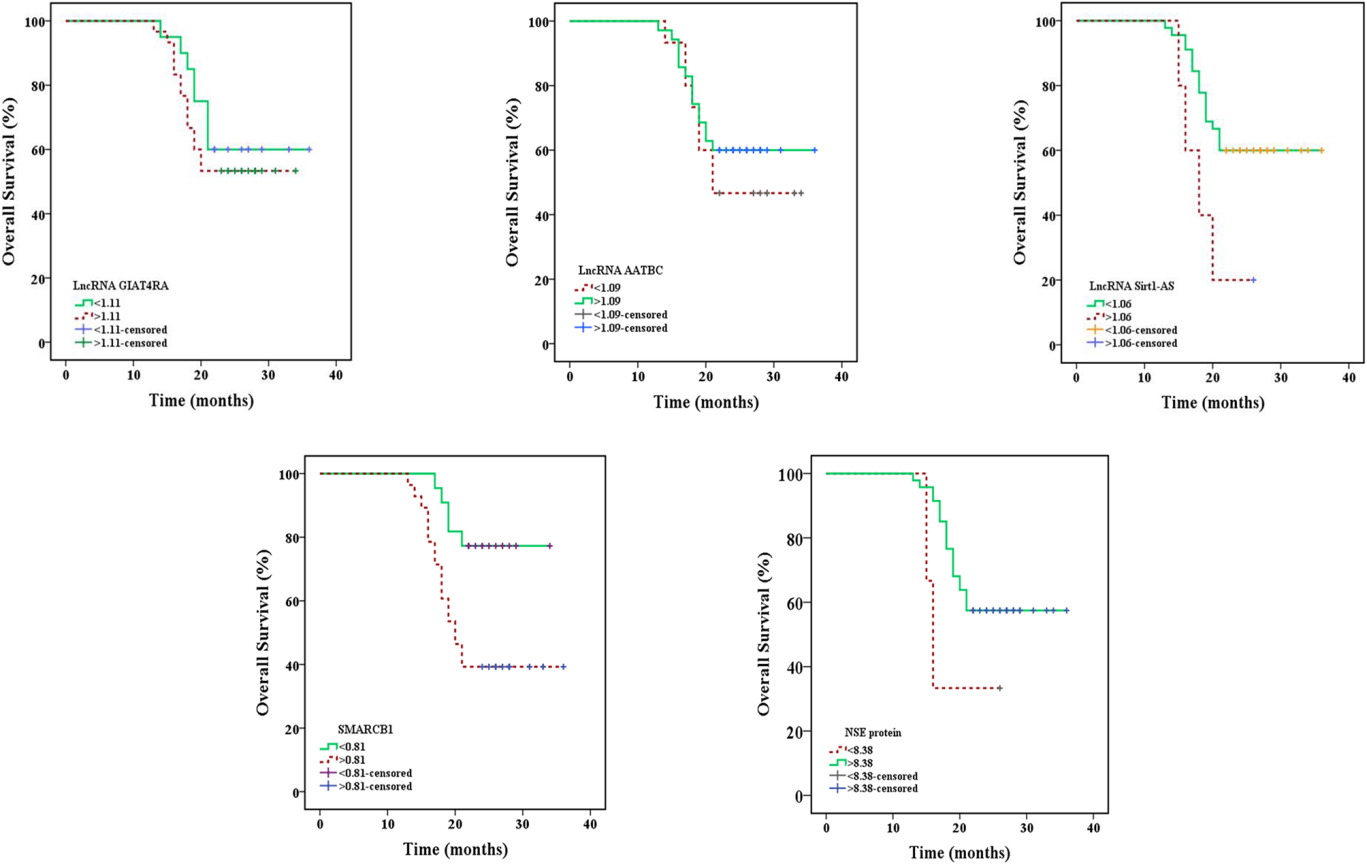
Kaplan-meier survival curves for overall survival of lncRNA GIAT4RA, lncRNA AATBC, lncRNA Sirt1-AS, SMARCB1 expression, and NSE in lung cancer patients

The analysis of survival curves based on follow-up data for patients with lung cancer revealed that statistically significant differences in DFS for individuals with greater lncRNA AATBC, and NSE than those with lower lncRNA AATBC expression, and NSE concentration (*P* = 0.036, 0.026 respectively) as well as for patients with lower lncRNA Sirt1-AS than those with higher expression (*P* = 0.014) (Table 4; Fig. 6). For overall survival, lncRNA Sirt1-AS and SMARCB1 showed significant differences between the two subgroups (*P* = 0.029, 0.006, respectively) (Table 5; Fig. 7).

**Table 4:** Disease Free Survival between high and low expression for lncRNA GIAT4RA, lncRNA AATBC, lncRNA Sirt1-AS, SMARCB1 expression, and NSE concentration in lung cancer patients

		Lung cancer (*n* = 50)	Metastatic n (%)	Non metastatic n (%)	Mean	Log-rank	
						χ^2^	P
LncRNA GIAT4RA	≤ 1.11	20	13 (65)	7 (35)	26.60	2.795	0.095
	> 1.11	30	26 (86.7)	4 (13.3)	23.56		
LncRNA AATBC	≤ 1.09	15	8 (53.4)	7 (46.7)	28.01	4.386	0.036*
	> 1.09	35	31 (88.6)	4 (11.4)	23.78		
LncRNA Sirt1-AS	≤ 1.06	45	35 (77.8)	10 (22.2)	25.27	6.037	0.014*
	> 1.06	5	4 (80)	1 (20.0)	20.00		
SMARCB1	≤ 0.81	22	17 (77.2)	5 (22.7)	26.05	0.751	0.386
	> 0.81	28	22 (78.6)	6 (21.4)	23.64		
NSE (ng/ml)	≤ 8.38	3	3 (100)		19.00	4.966	0.026*
	> 8.38	47	36 (76.6)	11 (23.4)	25.15		

*NSE* Neuron-specific enolase

**Table 5:** Overall survival between high and low expression for lncRNA GIAT4RA, lncRNA AATBC, lncRNA Sirt1-AS, SMARCB1 expression, and NSE concentration in lung cancer patients

		Lung cancer (*n* = 50)	Death n (%)	Survival n (%)	Mean	Log-rank	
						χ^2^	P
LncRNA GIAT4RA	≤ 1.11	20	8 (40)	12 (60)	29.10	0.543	0.461
	> 1.11	30	14 (46.7)	16 (53.3)	26.20		
LncRNA AATBC	≤ 1.09	15	8 (53.3)	7 (46.7)	25.60	0.503	0.478
	> 1.09	35	14 (40)	21 (60)	28.62		
LncRNA Sirt1-AS	≤ 1.06	45	18 (40)	27 (60)	28.77	4.775	0.029*
	> 1.06	5	4 (80)	1 (20)	19.00		
SMARCB1	≤ 0.81	22	5 (22.7)	17 (77.3)	30.54	7.667	0.006*
	> 0.81	28	17 (60.7)	11 (39.3)	24.78		
NSE (ng/ml)	≤ 8.38	3	2 (66.7)	1 (33.3)	19	2.319	0.128
	> 8.38	47	20 (42.6)	27 (57.4)	28.36		

*NSE* Neuron-specific enolase

## Discussion

Cellular DNA alterations are often minimal in most human disorders, but there are often noticeable alterations in gene expressions, which are linked to epigenetic disorders and further facilitate pathogen and disease progression [14]. One of the most important mechanisms of epigenetics is the regulation of non-coding RNAs (ncRNAs), which is independent of variations in DNA sequence [15, 16]. LncRNAs are an essential subclass of ncRNAs that act as biological regulators, responding to extracellular or intracellular signaling molecules to control downstream genes. Uncontrolled gene expression would result from dysregulation of lncRNAs [17]. The current results revealed a statistical up-regulation of lncRNA GIAT4RA and lncRNA AATBC and a significant down-regulation of lncRNA Sirt1-AS expression in all patients except for GIAT4RA in chronic inflammatory patients.

According to Yang et al.‘s findings, lncRNA GIAT4RA functions as a tumor suppressor and a regulator of ubiquitination in NSCLC. Upregulated lncRNA GIAT4RA did not affect chromatin modifier lymphoid-specific helicase (LSH) mRNA but reduced LSH protein levels by interfering with proteasome-mediated stabilization and deubiquitination by binding to its 227–589 amino acids. LSH is crucial for DNA methylation, enhances nucleosome density, and induces the stalling of RNA polymerase II [5, 18]. Ubiquitin C-Terminal Hydrolase L3 (UCHL3) stimulates LSH stabilization, and lncRNA GIAT4RA interferes with UCHL3-mediated LSH deubiquitination in a UCHL3-dependent manner [5].

On the contrary, the significant up-regulation of lncRNA GIAT4RA was indicated in the present study. This contradiction in results may be due to the stage at which samples were collected. The relative expression of genes within the transcriptome changes from stage to stage, some of which are only expressed at a particular stage. The obtained results further confirmed that lncRNA GIAT4RA expression was significantly related to the stage of lung cancer. The expression of lncRNA GIAT4RA trended to upregulate with the stage of lung cancer.

MiRNA sponging is an important regulatory mechanism of lncRNAs. Through the sponging of miRNA, lncRNA AATBC may act as a competitive endogenous RNA (ceRNA) to influence the downstream target genes [19]. In nasopharyngeal carcinoma (NPC), lncRNA AATBC was significantly expressed and upregulated desmosome-associated protein pinin (PNN) expression by miR-1237-3p sponging. LncRNA AATBC may function as a molecular sponge for assisting miR-1237-3p bind to PNN. Consequently, PNN interacted with the EMT activator zinc finger E-box binding homeobox 1 (ZEB1) and increased the expression to induce EMT in NPC cells [7]. Since miR-1237-3p is expressed in lung cancer [20], therefore, lncRNA AATBC may function as ceRNA of PNN gene by competitively interacting with miR-1237-3p and eventually increasing lung cancer via miR-1237-3p/PNN/ZEB axis.

In breast cancer, lncRNA AATBC functions as a Y-box binding protein 1 (YBX1) scaffold and controls Hippo signaling through lncRNA AATBC/YBX1/mammalian sterile 20 like kinase 1 (MST1) axis [21]. LncRNA AATBC was shown to regulate several components in the Hippo signaling pathway. In some tumor cells, MST1/2 and LATS1/2 may prevent phosphorylation of YAP1, causing its translocation to the nucleus, where it functions as a transcription factor that promotes proliferation, invasion, and metastasis [22]. LncRNA AATBC and YBX1 may interact with each other, and YBX1 was also discovered to interact with MST1. As a result, lncRNA AATBC may act as a molecular guide to facilitate the binding between YBX1 and MST1. In addition, by forming the YBX1/MMST1 complex, lncRNA AATBC may take part in the YBX1-mediated suppression of YAP1 phosphorylation [21]. A previous study showed that YBX1 is highly expressed in numerous cancers, such as lung cancer [23], and the Hippo pathway has a role in lung carcinogenesis [24]. Consequently, lncRNA AATBC may promote lung cancer migration by activating the Hippo pathway through the lncRNA AATBC/YBX1/MST1 axis.

LncRNAs can influence mRNA stabilization by interacting directly with miRNA or RNA binding proteins (RBP) binding sites in target mRNA, sequestering miRNAs or RBPs to prevent their binding to mRNA, serving as scaffolds to improve RBP-mRNA binding, or modifying N6-methyladenosine (m6A) levels of target mRNAs by interacting with the m6A machinery [25]. The mammalian genome frequently contains natural antisense transcripts, which control gene expression at both transcriptional and post-transcriptional levels. One of the essential post-transcriptional regulation processes that influence the sense transcript in a locus-specific manner is targeting the sense by its antisense RNA transcript [26].

As a tumor suppressor gene, Sirt1 has crucial roles in maintaining heterochromatin structure by deacetylating histones and in regulating Rad5, 1γH2AX, Brca1, and NBS1 foci formation, which are involved in cell cycle checkpoints and DNA damage repair [27]. Sirt1 protein and mRNA levels were considerably reduced by down-regulating lncRNA Sirt1-AS expression [28].

By reducing lncRNA Sirt1-AS, the expression of fibronectin1, α-smooth muscle actin, and collagen1 was upregulated, whereas the expression of E-cadherin was downregulated, resulting in increased EMT. Moreover, lncRNA Sirt1-AS was a negative regulator of EMT and has an anti-fibrosis function, where it inhibited TGF-β1-induced EMT of alveolar epithelial cells. LncRNA antisense/mRNA duplex may cover miRNA binding sites [28]. LncRNA Sirt1-AS relieved Sirt1 transcriptional repression through competition with miR-34a and miR-22 [29, 30]. Since miR-34a expression is downregulated in lung cancer patients [31], lncRNA Sirt1-AS may block the miR-34a-binding site on Sirt1, thereby inhibiting EMT in lung cancer.

The insignificant difference in SMARCB1 expression in lung patients is consistent with previous studies that indicated that SMARCB1 mutation was frequently observed in pancreatic cancer, gastrointestinal cancer, and sinonasal carcinoma, but it was seldom recorded in lung carcinoma [32, 33]. According to Herpel et al., SMARCA4 and SMARCA2 were shown to be defective in NSCLC, while SMARCB1 expression was found to be intact in all cases of NSCLC patients [34]. Moreover, SMARCB1 downregulation seems to be an uncommon finding in primary lung cancer, as well as the immunophenotypic and morphological resemblance to SMARCB1-deficient carcinoma of the sinonasal tract, indicates that pulmonary SMARCB1-deficient carcinoma could be a distinct diagnostic entity. Surprisingly, SMARCB1 is crucial for survival in many cancer cell lines, despite being a tumor suppressor gene [35]. Additionally, synovial sarcoma is characterized by a decreased level of SMARCB1 protein and high expression of SMARCB1 mRNA, which point to a post-transcriptional involvement in SMARCB1 degradation [36].

Lung cancer is a malignant condition that is linked to neuroendocrine differentiation [37]. NSE is not secreted in a normal state. When axons are damaged, NSE is elevated to restore homeostasis. NSE is a traditional biomarker that assesses functional damage to neurons. A higher NSE concentration in tissues and circulation is possibly associated with the malignant proliferation of neuroendocrine tissues, suggesting that it may be useful for cancer staging, diagnosis, and therapy planning [38].

The current findings showed dysregulation of lncRNA AATBC, lncRNA Sirt1-AS, and SMARCB1 expression in patients with inflammatory diseases. Alterations in lncRNA expression, which exhibit significantly more specific cell-type expression patterns than mRNAs, may contribute to chronic respiratory diseases. Furthermore, it was found that disease-associated lncRNAs show significantly more variation in expression than disease-associated mRNAs, and as a result, lncRNAs are thought to be possible biomarkers. Finding lncRNAs linked to diseases or disease endotypes can also aid in knowing the patho-mechanism of these diseases [39]. Cigarette smoke causes oxidative stress, which is a known risk factor for chronic obstructive pulmonary disease (COPD), which accounts for 90% of COPD-related deaths. In response to cigarette smoke, some lncRNA transcripts are upregulated while others are downregulated [40]. Particularly, lncRNA is upregulated in lung cancer, indicating a link between smoking, COPD, and the growth of lung cancer [41]. The present study confirms the significant correlations between all studied genes and smoking in patients with chronic inflammatory disease.

SMARCB1 plays a significant role in inflammation-associated genes. SMARCB1 is a key regulator of the immune response and cell cycle, where SMARCB1 was found to bind to IL6 promoter in a steady state and to dissociate during an active immune response state, indicating that it was a direct IL6 repressor. Additionally, the loss of SMARCB1 function changes the formation and operation of SWI/SNF complexes, which results in a changed cellular gene expression profile [42].

ROC curves demonstrated the viability of using lncRNA GIAT4RA, lncRNA AATBC, and lncRNA Sirt1-AS as diagnostic biomarkers compared with NSE for lung cancer patients. NSE protein is better than lncRNA Sirt1-AS, lncRNA AATBC, and lncRNA GIAT4RA. In chronic inflammatory patients, lncRNA AATBC, lncRNA Sirt1-AS, and SMARCB1 could be used as biomarkers where lncRNA Sirt1-AS is superior to SMARCB1 and lncRNA AATBC. The survival analyses showed that the poorer DFS and OS of lung cancer patients were linked to reduced lncRNA Sirt1-AS expression. Furthermore, poor survival in patients with lung cancer was indicated by high expression of lncRNA AATBC, SMARCB1, and concentration of NSE.

## Conclusion

LncRNA GIAT4RA, lncRNA AATBC, and lncRNA Sirt1-AS were differentially expressed in patients with lung and those with chronic inflammatory diseases. The resulting altered expression of lncRNAs could play a significant role in the development and progression of lung cancer diseases. LncRNAs could be a potential target for novel treatments to reduce mortality in lung cancer patients.

LncRNA GIAT4RA, lncRNA AATBC, and lncRNA Sirt1-AS might function as diagnostic biomarkers. LncRNA AATBC, lncRNA Sirt1-AS, SMARCB1, and NSE could be valuable prognostic biomarkers for lung cancer patients. Even so, extensive clinical cohort studies are advised to confirm the functions of selected lncRNAs.

## Electronic supplementary material

Below is the link to the electronic supplementary material.

Supplementary Material 1

## Data Availability

All the data analyzed during this study is included in the article.

## Abbreviations

CELIA: Electrochemiluminescence immunoassay
DFS: Disease Free Survival
GAPDH: Glyceraldehyde 3-phosphate dehydrogenase
LncRNAs: Long noncoding RNAs
NSE: Neuron-specific enolase
OS: Overall Survival
ROC: Receiver operating characteristic curve
RT-PCR: Real time-Polymerase Chain Reaction
SMARCB1: Switch/sucrose non-fermentable (SWI/SNF)-related matrix-associated actin-dependent regulator of chromatin, subfamily B, member 1
