# Supplementary material for: Expression profiling of circulating lncRNA GIAT4RA, lncRNA AATBC, lncRNA Sirt1-AS, and SMARCB1 in lung cancer patients
Source: BMC Cancer. 2024 Sep 23;24:1175. doi: 10.1186/s12885-024-12896-1 (PMC11421180; doi:10.1186/s12885-024-12896-1)
Supplement: Supplementary file 1 — Supplementary Material 1 [file 12885_2024_12896_MOESM1_ESM.docx]

**
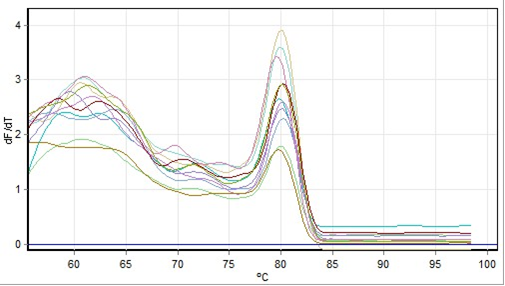
**


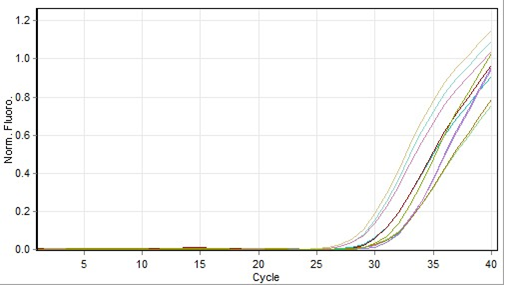


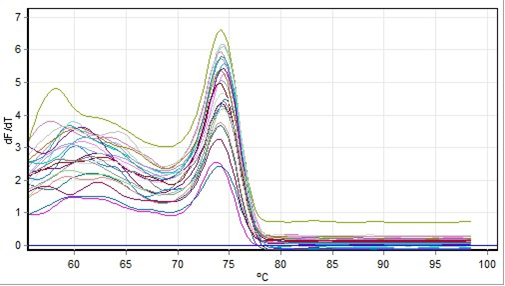


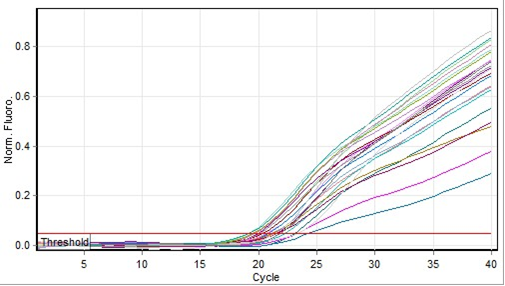


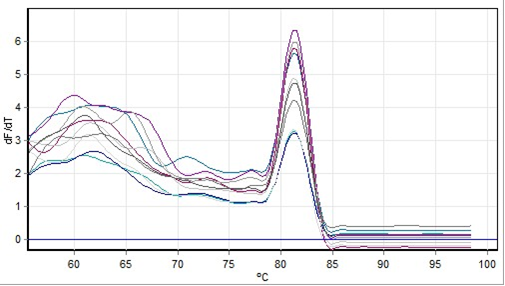


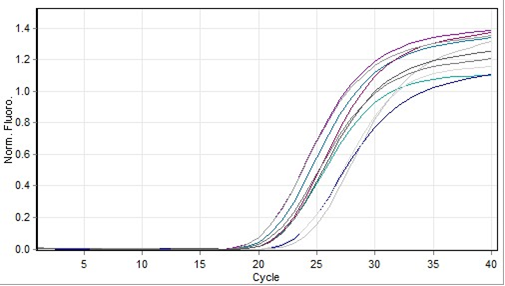


**AA**

**C**

**B**


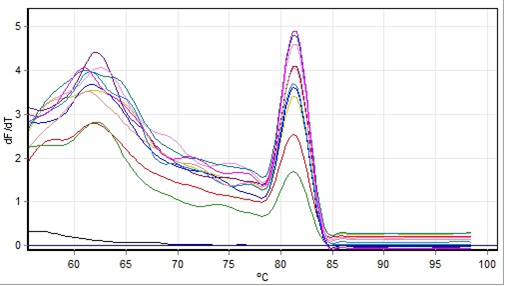


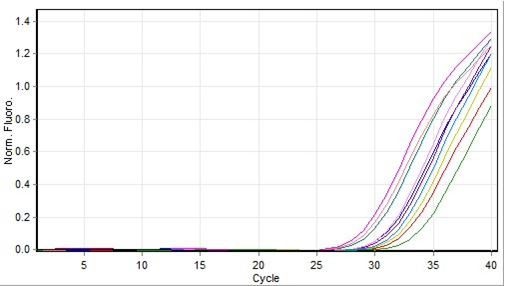


**
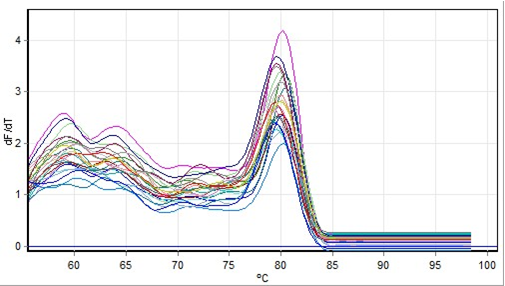
**


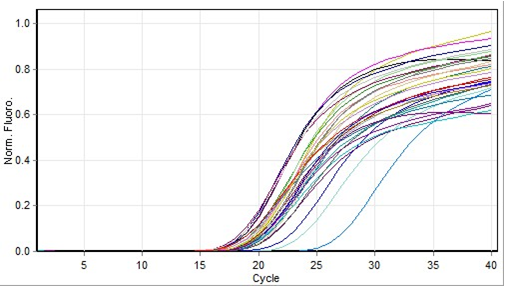


**E**

**D**

Figure 1: Melting and amplification curves for (A) lncRNA GIAT4RA, (B) lncRNA AATBC, (C) lncRNA Sirt1-As, (D) SMARCB1, and (E) GAPDH.

Table 1: Correlation of CEA with lncRNA GIAT4RA, lncRNA AATBC, lncRNA Sirt1-AS, SMARCB1, NSE in patients with chronic inflammatory disease and lung cancer patients.

|  | **CEA** | | | |
| --- | --- | --- | --- | --- |
|  | **Chronic inflammatory patients** | | **Lung cancer patients** | |
|  | r_s_ | P | r_s_ | P |
| **LncRNA GIAT4RA** | -0.174 | 0.358 | 0.020 | 0.889 |
| **LncRNA AATBC** | 0.488 | 0.006* | 0.234 | 0.103 |
| **LncRNA Sirt1-AS** | 0.024 | 0.902 | 0.190 | 0.187 |
| **SMARCB1** | 0.081 | 0.677 | -0.119 | 0.410 |
| **NSE** | -0.107 | 0.574 | 0.102 | 0.479 |

r_s_: Spearman coefficient *: Significant at P ≤ 0.05

Table 2: Relation of lncRNA GIAT4RA, lncRNA AATBC, lncRNA Sirt1-AS, SMARCB1, NSE with patients' characteristics.

|  | **N** | **LncRNA GIAT4RA** | **LncRNA AATBC** | **LncRNA Sirt1-AS** | **SMARCB1** | **NSE** |
| --- | --- | --- | --- | --- | --- | --- |
|  | **Chronic inflammatory patients** | | | | | |
| **Gender**  **Male**  **Female**  **U (P)** | **21**  **9** | 0.97 ± 0.59  1.15 ± 0.75  U=33.0 (P=0.01*) | 2.14 ± 0.47  5.58 ± 2.63  U=46.0 (P=0.049*) | 0.62 ± 0.31  0.05 ± 0.01  U=24.0 (P=0.019*) | 0.79 ± 0.38  0.72 ± 0.02  U=49.0 (P=0.15) | 4.47 ± 2.83  3.49 ± 2.19  U=73.0 (P=0.349) |
| **Smoking**  **Yes**  **No**  **U (P)** | **22**  **8** | 0.998 ± 0.60  1.38 ± 0.80  U=45.0 (P=0.044*) | 6.11 ± 0.79  2.02 ± 0.47  U=37.0 (P=0.017*) | 0.62 ± 0.16  0.54 ± 0.04  U=29.0 (P=0.038*) | 1.80 ± 0.79  0.06 ± 0.02  U=36.0 (P=0.037*) | 4.36 ± 2.81  3.66 ± 2.27  U=74.0 (P=0.534) |
|  |  | **Lung cancer patients** | | | | |
| **Gender**  **Male**  **Female**  **U(P)** | **44**  **6** | 2.42±0.42  2.54 ± 0.71  U=215 (P=0.780) | 5.58 ± 0.83  2.26 ± 0.91  U=116 (P=0.011*) | 0.47 ± 0.09  0.44 ± 0.11  U= 214 (P=0.750) | 2.29 ± 0.48  3.85 ± 1.00  U=175 (P=0.233) | 17.32 ± 3.28  14.11 ± 6.10  U=81.50 (P=0.134) |
| **Age**  **>40**  **<40**  **U(P)** | **47**  **3** | 2.38 ± 0.37  3.55 ± 1.04  U=35.0 (P=0.012*) | 4.77 ± 0.72  4.84 ± 2.55  U=97.0 (P=0.616) | 0.45 ± 0.08  0.60 ± 0.41  U=112 (P=0.987) | 2.49 ± 0.43  5.41 ± 2.89  U=65.0 (P=0.125) | 16.97 ± 3.91  16.33 ± 0.19  U=47 (P=0.364) |
| **Smoking**  **Yes**  **No**  **U(P)** | **44**  **6** | 2.24 ± 0.37  4.00 ± 0.98  U=63.0 (P=0.039*) | 5.04 ± 0.76  2.88 ± 1.40  U=98.0 (P=0.310) | 0.47 ± 0.08  0.38 ± 0.21  U=109 (P=0.492) | 2.21 ± 0.42  6.04 ± 1.47  U=62.0 (P=0.037*) | 17.32 ± 3.28  14.11 ± 6.10  U=81.50 (P=0.134) |
| **Family history**  **Negative**  **Positive**  **U(P)** | **36**  **14** | 2.15 ± 0.41  3.22 ± 0.7  U=161.0 (P=0.049*) | 5.05 ± 1.15  4.10 ± 1.02  U=247.0 (P=0.914) | 0.55 ± 0.09  0.23 ± 0.07  U=130.0 (P=0.009*) | 2.58 ± 0.50  2.88 ± 0.96  U=242.0 (P=0.829) | 16.88 ± 2.56  17.06 ± 6.03  U=249.50(P=0.957) |
| **Tumor size**  **<5**  **>5**  **U(P)** | **16**  **34** | 1.49 ± 0.38  2.79 ± 0.45  U=174.0 (P=0.144) | 3.10 ± 1.08  5.37 ± 0.84  U=151.0 (P=0.048*) | 0.50 ± 0.08  0.45 ± 0.10  U=165.0 (P=0.095) | 2.69 ± 0.83  2.66 ± 0.53  U=238.0 (P=0.965) | 17.59 ± 2.65  16.62 ± 4.22  U=217 (P=0.253) |
| **Lymph node metastasis**  **Negative**  **Positive**  **U(P)** | **8**  **42** | 0.97 ± 0.54  2.69 ± 0.39  U=76.0 (P=0.037*) | 1.55 ± 1.02  5.31 ± 0.76  U=63.0 (P=0.013*) | 0.44 ± 0.10  0.46 ± 0.08  U=122.0 (P=0.442) | 1.43 ± 0.67  2.87 ± 0.50  U=111.0 (P=0.282) | 17.05 ± 1.09  16.91 ± 4.12  U=150.50(P=0.649) |
| **Stage**  **II +III**  **IV**  **U(P)** | **10**  **40** | 0.98 ± 1.25  2.79 ± 2.60  U=102.5 (P=0.018*) | 2.90 ± 4.25  5.86 ± 5.20  U=111.5 (P=0.030*) | 1.98 ± 2.6  2.73 ± 3.2  U=164.5 (P=0.395) | 16.79 ± 1.23  16.96 ± 4.2  U=197.5 (P=0.952) | 16.7 ± 1.24  16.96 ± 4.20  U=197.5 (P=0.952) |
| **Grade**  **I+II**  **III**  **U(P)** | **27**  **23** | 2.54 ± 0.51  2.34 ± 0.50  U=305.0 (P=0.922) | 5.64 ± 0.92  3.77 ± 0.82  U=267.0 (P=0.397) | 0.62 ± 0.12  0.283 ± 0.08  U=160.0 (P=0.004*) | 3.34 ± 0.60  1.88 ± 0.64  U=192.0 (P=0.021*) | 17.14 ± 2.13  16.67 ± 5.14  U=259.5 (P=0.321) |

The results were expressed mean ± S.D U: Mann Whitney test P: P value for comparing between different categories.
